# From repair to right heart failure: Understanding the evolving landscape in tetralogy of fallot

**DOI:** 10.1007/s10741-025-10583-2

**Published:** 2025-12-06

**Authors:** Nunzia Borrelli, Ferdinando Carlo Sasso, Berardo Sarubbi

**Affiliations:** 1 Adult Congenital Heart Disease Unit, AO Dei Colli - Monaldi Hospital, Napoli, 80131 Italy; 2 University of Campania “Luigi Vanvitelli”, Naples, Italy; 3 Department of Advanced Medical and Surgical Sciences, University of Campania “Luigi Vanvitelli”, Naples, Italy; 4 Parthenope University of Naples, Naples, Italy

**Keywords:** Tetralogy of fallot, Heart failure, Right ventricle, Congenital heart disease

## Abstract

Surgical repair and clinical management have significantly improved Tetralogy of Fallot (TOF) patient survival rates. Despite these advancements, adults with repaired TOF remain at high risk for long-term complications, particularly heart failure, which constitutes the primary cause of mortality in this population. This review synthesizes current evidence on right heart failure in repaired TOF, with emphasis on pathophysiological mechanisms, diagnostic strategies, and management approaches. The chronic hemodynamic burden of residual lesions and surgical sequalae initiates a maladaptive cascade that results in progressive right ventricular dysfunction and, ultimately, clinical manifestations of right heart failure. The discussion includes the role of multimodal imaging, electrocardiographic monitoring, and exercise testing, as well as the limited evidence for conventional pharmacological therapies in patients with repaired TOF and right heart failure. This review aims to serve as a comprehensive resource to guide clinical decision-making and to highlight key knowledge gaps that warrant further research to improve long-term outcomes in this patient group.

## Introduction

Tetralogy of Fallot (TOF) is a surgical successful story, however, adult life chapter of these patients is anything but risk-free. TOF is the most prevalent form of cyanotic congenital heart disease (CHD), with an estimated occurrence rate of approximately 32.6 cases for every 100,000 live births. Although surgical repair and medical treatment have significantly improved survival rates, enabling patients to reach adulthood, these individuals continue to face life-threatening cardiovascular complications throughout their lives [1, 2].

Indeed, patients with TOF present an increased incidence of heart failure (HF), ventricular arrhythmias, and sudden cardiac death. Notably, HF is recognized as the predominant cause of mortality in adult patients with TOF [3–5].

**Fig. 1:**
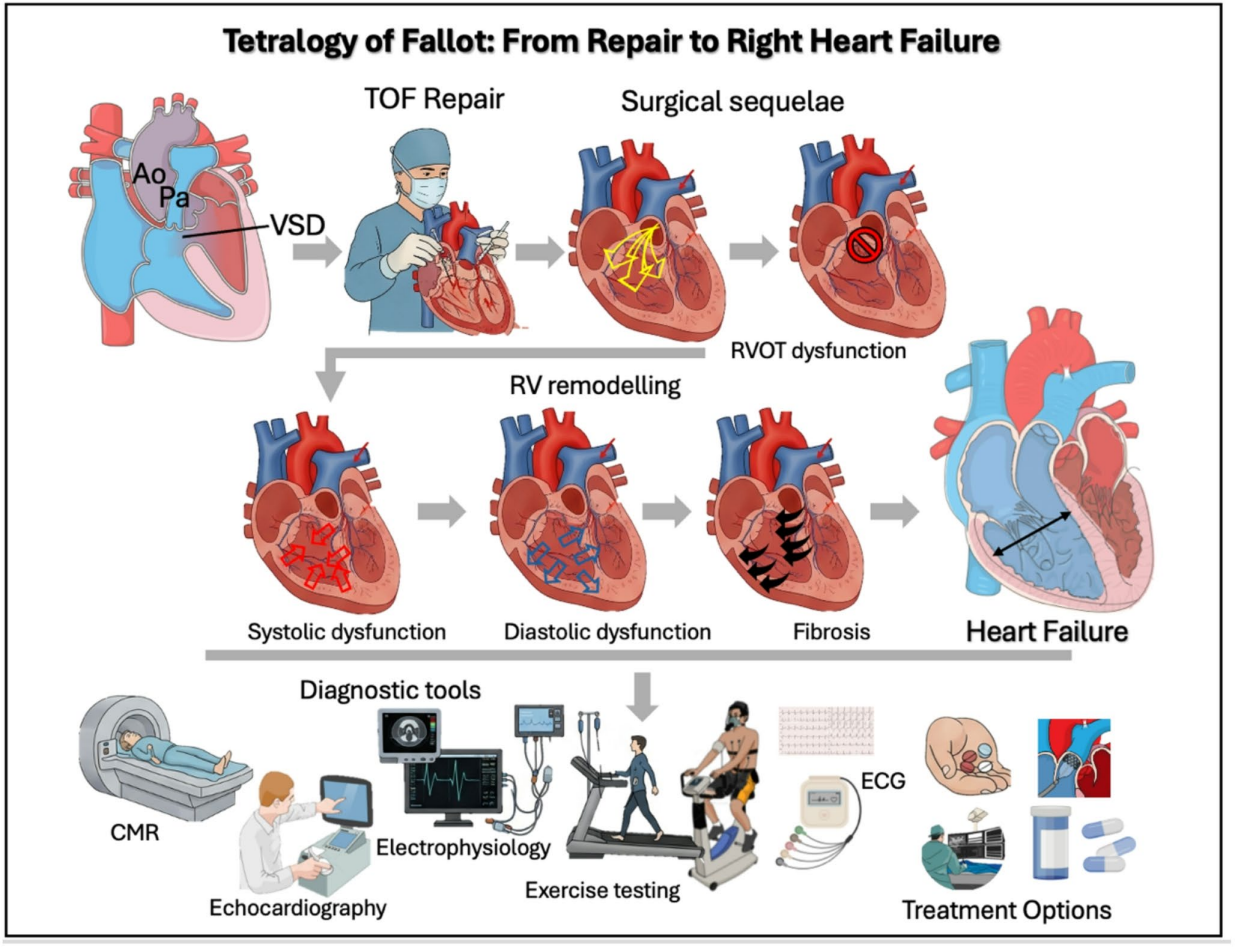
Pathophysiological mechanisms, diagnostic strategies, and management approaches of right heart failure in patients with Tetralogy of Fallot

The insidious process leading to HF can appear many years after the initial corrective surgery. Chronic hemodynamic burdens from residual lesions – especially pulmonary regurgitation and right ventricular outflow tract (RVOT) obstruction - starts a cascade of maladaptive right ventricle (RV) remodelling. This eventually leads to functional decline and the clinical syndrome of right heart failure (RHF) [6] (Fig. 1). Despite advances in the management of left HF, the approach to RV dysfunction in repaired TOF remains fragmented and lacks a well-defined clinical consensus, representing a major cause of long-term morbidity and mortality.

This review argues that improving clinical outcomes requires an integrated approach, focused on a personalized understanding of RV dysfunction through multimodal imaging, risk stratification, and the implementation of emerging therapies.

## Pathophysiology of right heart failure in repaired TOF

### Right ventricular outflow tract dysfunction

The pathophysiology of RHF in repaired TOF (rTOF) patients recognizes multiple determinants. Surgical repair of TOF often involves disrupting the pulmonary valve to relieve RVOT obstruction, typically requiring a transannular patch. This can lead to residual pulmonary regurgitation, a key factor contributing to RHF [7].

In the initial postoperative period, pulmonary regurgitation is often well tolerated due to reduced RV compliance, low pulmonary artery capacitance, and elevated heart rates that shorten diastolic duration. Over time, however, chronic pulmonary regurgitation, frequently accompanied by residual RVOT obstruction, can lead to progressive RV overload. This overload may result in RV dilation, worsening pulmonary regurgitation, and ultimately a decompensated state characterized by a reduced RV mass-to-volume ratio, increased wall stress, and RV dysfunction [6, 8].

Moreover, the placement of an outflow patch during surgical repair frequently worsens RV contractility, resulting in RVOT akinesia or dyskinesia, outflow patch aneurysm, RV free wall fibrosis, and conduction delay [8, 9]. Also, the resection of extensive muscular bands and ischemic damage at the time of surgery may contribute to dysfunction of the RVOT [10].

Persistent or recurrent RVOT obstruction is common following surgical correction, but its impact is complex and not completely understood. Some researchers suggest that residual RVOTO may provide some degree of protection against pulmonary regurgitation and RV remodelling, leading to more favourable RV dimensions and degrees of pulmonary regurgitation [11, 12]. However, other studies indicate that elevated RV pressure is associated with reduced biventricular strain, does not prevent the need for pulmonary valve replacement, and acts as an independent predictor of unfavourable outcome [13, 14].

### Right ventricle diastolic dysfunction

Right ventricular diastolic dysfunction can occur early in patients with TOF and constitutes an initial factor contributing to ventricular dysfunction.

Normal diastolic function necessitates adequate preload and minimal myocardial stiffness [9]. While pulmonary regurgitation often keeps preload stable in TOF patients, invasive data show that at least half of TOF patients present elevated RV end-diastolic pressures [15, 16]. Indeed, in these patients, myocardial stiffness may be exacerbated by pressure overload with reactive interstitial fibrosis or by an intrinsic cardiomyocyte stiffness [9].

The identification of diastolic dysfunction remains challenging because reliable echocardiographic markers are currently lacking.

The presence of end-diastolic forward flow in the pulmonary artery has been referred to as RV restrictive physiology in TOF patients and is believed to indicate a potential decrease in RV diastolic compliance. This pattern is classically identified with Doppler echocardiography as forward flow in the pulmonary artery in late diastole along with premature opening of the pulmonary valve during atrial systole [17]. While it has been associated with a longer healing period following surgery [18], its significance on long-term follow-up is questionable [19, 20]. In a more recent study by Egbe et al., inferior vena cava dilatation and hepatic vein diastolic flow reversal had the best sensitivity and specificity for detecting severe RV diastolic dysfunction at cardiac catheterization and were an independent risk factor for death/transplant in the third decade of life of these patients [16]. The role of right atrial function is a promising but still poorly investigated parameter in the assessment of RV dysfunction. In rTOF, right atrium reservoir, conduit, and pump function are diminished and appear to correlate with RV systolic and diastolic dysfunction [21] and exercise functional capacity [22]. In a study by Luijnenburg et al., TOF patients with enlarged right atrium and abnormal emptying function were demonstrated to have higher NT-proBNP level, a lower exercise capacity, and abnormal diastolic RV function [23]. Lastly, the presence of right atrium enlargement before pulmonary valve replacement was correlated with a worse outcome after replacement, demonstrating a higher incidence of death, aborted sudden cardiac death, ventricular arrhythmias, worsening HF class, or sustained supraventricular tachycardia [24].

### Right ventricle remodelling: adaptive and maladaptive changes in RV structure and function

In response to increased preload related to pulmonary regurgitation, the RV undergoes a progressive dilation, initially preserving the ejection volume and forward flow [25]. During this compensatory phase, the RV preserves near-normal systolic function and cardiac output, resulting in a prolonged asymptomatic period. When compensatory mechanisms fail, the mass-to-volume ratio decreases, end-systolic volume increases, and ejection fraction declines [8].

The most common maladaptive histological features in TOF patients after repair are myocyte hypertrophy and myocardial fibrosis. The degree of hypertrophy increases with age, most likely as an adaptive response initially, until the maladaptive trait emerges [26]. The myocytes present an altered orientation, having a more oblique orientation and a well-represented mid-layer of circumferential fibres compared to normal controls. This hypertrophy is also not homogeneous, being more represented in the infundibulum compared to other parts of the RV [27].

Myocardial fibrosis has been demonstrated in different sites of patients with repaired TOF, including the RVOT, ventricular septal defect patching, RV inferior insertion point, apex, left ventricle (LV) inferior, and lateral wall [2]. Cardiac magnetic resonance (CMR) studies demonstrated an increased late gadolinium enhancement (LGE) - showing increased cardiac fibrosis - correlates with exercise intolerance, malignant tachyarrhythmias, and sudden cardiac death in repaired ToF [28–30]. Furthermore, as shown by Ghonim et al., 3D LGE has been revealed to be a more powerful predictor of inducible ventricular tachycardia during invasive programmed electrical stimulation than well-known predictors such as age, QRS duration >180 ms, and non-sustained VT.

Furthermore, the presence of diffuse fibrosis, demonstrated by an increased extracellular volume fraction (ECV) on CMR T1 mapping, has been shown to be related to adverse events in the TOF population. In a study by Chen et al., an increased ECV was associated with RV volume overload and arrhythmias in adults with repaired TOF (median age 23.3 years) [31]. LV and RV ECV values were positively correlated, suggesting interventricular interaction. However, in this study, LV ECV above the upper limit of normality (>28%), and not RV ECV, was associated with RV volume overload and arrhythmia [31]. On the contrary, a more recent study by Shiina et al. demonstrated RV ECV and septal ECV can predict cardiac events (cardiac surgery, cardiac death, HF requiring hospitalization, ventricular arrhythmias requiring medication, and ablation and/or implantation of a cardiac defibrillator) in symptomatic adult patients (mean age 35.4 ± 13.8 years) with rTOF [32]. Finally, shorter T1 values have been demonstrated after pulmonary valve replacement, suggesting diffuse fibrosis may improve after treatment [33], although these values have not been shown to completely normalize [34].

## Clinical manifestations and diagnosis

### ECG, holter, and invasive electrophysiology study

Arrhythmias and sudden cardiac death are common complications in TOF patients even after an optimal repair. The mechanisms underlying arrhythmias in this population often differ from those observed in patients with acquired heart disease. Most arrhythmias in TOF are caused by macroreentrant circuits, which may be the result of either repair techniques or the underlying anatomy. Progressive hemodynamic events, such as RV pressure or volume overload, can cause atrial and ventricular remodelling, which affects conduction and may predispose patients with high-risk anatomical substrates to developing clinical arrhythmias [35].

Arrhythmia risk assessment includes a comprehensive review of the patient’s medical history (e.g., palpitations, syncope), a 12-lead ECG, rhythm monitoring (including an implanted loop recorder when indicated), and, in selected cases, an invasive electrophysiological study.

In a multicenter study of 556 adult patients with rTOF, 43.3% presented with a sustained arrhythmia or arrhythmia intervention, 20.1% of which were atrial tachyarrhythmias. While intraatrial reentrant tachycardia was associated with right atrial enlargement, hypertension, and the number of cardiac surgeries, atrial fibrillation was associated with older age, lower LV ejection fraction, left atrial dilation, and the number of cardiac surgeries. Ventricular arrhythmias were present in 14.6% of cases and associated with the number of cardiac surgeries, QRS duration, and LV diastolic dysfunction [36].

Khairy et al. [37] identified six weighted clinical variables associated with an increased risk of appropriate ICD shocks in a population of patients with primary prevention repaired TOF, including the presence of a prior palliative shunt, inducible sustained ventricular tachycardia, QRS duration ≥ 180 milliseconds (ms), ventriculotomy incision, non-sustained ventricular tachycardia, and LV end-diastolic pressure ≥ 12 mmHg. However, calculating this risk score requires invasive assessment of ventricular pressure and an electrophysiological study, limiting its use as a screening tool.

The PREVENTION-ACHD (Prospective Study of Implantable Cardioverter-Defibrillator Therapy and Sudden Cardiac Death in Adults with Congenital Heart Disease) risk score was developed to identify high-risk characteristics for sudden cardiac death in patients with CHD. The seven risk factors identified included coronary artery disease, New York Heart Association (NYHA) class II/III, sustained ventricular tachyarrhythmias, LV ejection fraction < 40%, RV ejection fraction < 40%, QRS duration ≥ 120 ms, and QT dispersion ≥ 70 ms. TOF patients with four risk factors had a 3% annual risk of sudden cardiac death, while TOF patients with six risk factors had a 14% annual risk [38].

The 2020 European Society of Cardiology Guidelines for Adult Patients with CHD [39] recommends invasive electrophysiological study with programmed electrical stimulation for risk stratification in patients with additional risk factors (LV/RV dysfunction, non-sustained symptomatic ventricular tachyarrhythmias, QRS duration ≥ 180 ms, extensive RV scarring on CMR).

### Exercise testing

In patients with rTOF, reduced exercise capacity is a common and potentially debilitating morbidity. The main factors limiting exercise capacity in patients with rTOF are still unclear due to the complex interaction underlying progressive exercise intolerance. However, several variables have been implicated, including RV and LV function, severity of respiratory failure, advanced age at the time of total repair, age at cardiopulmonary exercise testing, the presence of residual shunt, pulmonary arterial hypertension, peak heart rate, and indexed LV and RV end-diastolic volumes [40]. Although exercise performance in patients with repaired TOF is thought to decline with age, a large metanalysis of 2239 TOF patients undergoing cardiopulmonary exercise testing found no difference in exercise performance in different age groups, suggesting that young patients are equally affected by functional limitation [40].

Reduced peak oxygen consumption (peak VO2), increased minute ventilation to carbon dioxide production (VE/CO2) ratio, and abnormal heart rate response have been associated with morbidity and mortality [41–44]. In particular, a peak VO2 < 27 mL/kg/min [45] and a value of VE/VCO2 ​>​31 [46] have been associated with an increased risk of major adverse cardiac events. While the ideal cutoff for freedom from death or ventricular arrhythmia at five years was found to be a peak predicted VO2 cutoff of 62% (sensitivity 82% and specificity 63%) [46].

### Echocardiography

Evaluating RV dimension and function with echocardiography may be challenging, particularly in the context of complex CHD. Speckle-tracking and 3-dimensional (3D) echocardiography have been shown to be a reliable technique for evaluating the RV without geometric assumptions. Additionally, speckle-tracking can be used to evaluate RV function independently of acute preload variations [47].

3D echocardiography showed good correlation with CMR in the assessment of right ventricular volumes, although with a known slight underestimation, and of ejection fraction [48]. 3D echocardiographic data showed that the right ventricular shape of patients with repaired TOF and severe pulmonary insufficiency differs from that of healthy controls and changes throughout the cardiac cycle. Indeed, they have a flatter RV free wall with a more rounded apex, and a more convex RVOT and interventricular septum. During the cardiac cycle, from end-diastole to end-systole, the mid-RV free wall and interventricular septum become less convex, while the convexity of the apical free wall increases during end-systole. Moreover, in the same study, a more convex RVOT at end-systole was associated with impaired RV systolic function in rTOF [49].

Longitudinal strain has been demonstrated to be a sensitive marker of early deterioration in RV performance and to predict low functional capacity in patients with rTOF [50, 51]. Van Grootel et al. demonstrated that, in patients with rTOF, longitudinal deformation of the RV free wall was markedly reduced in patients who have died or experienced HF and was associated with adverse cardiac events [52].

Although longitudinal deformation parameters showed less load dependency compared with conventional echocardiographic indices, they are still affected by afterload. Non-invasive myocardial work has been proposed to overcome the effect of loading conditions on traditional and advanced echocardiographic systolic function indices [53]. Similar to the assessment for the LV, myocardial work indices can also be calculated for the RV, using the opening and closing of the pulmonary and tricuspid valves as cardiac cycle times and noninvasively deriving the instantaneous right ventricular pressure from the pulmonary artery systolic pressure [54]. The role of right ventricular myocardial work indices in TOF is still unknown, but they may represent an interesting topic for future research, given their correlation with invasive measures of right ventricular systolic function and prognostic value, particularly in patients with pulmonary hypertension [54].

In addition to right ventricular dysfunction, longitudinal, radial, and circumferential strain of the LV have been shown to be compromised, despite maintaining a normal ejection fraction, potentially through a mechanical interdependence between the two ventricles. The circumferential radial component appeared to be the most affected [55]. LV torsion has also been shown to be impaired, with basal rotation being reversed (counterclockwise) and apical rotation initially compensatorily enhanced at younger ages, then becoming impaired at older ages [55–57].

Lastly, several studies have found that both the left and right atrial mechanics are impaired in this population. In particular, in patients with rTOF, a history of potentially fatal arrhythmias has been associated with abnormal left and right atrial strain [58, 59].

### CMR and computed tomography

CMR is particularly suited for evaluating patients with rTOF due to its capacity to characterize residual lesions, assess hemodynamic sequelae, facilitate risk stratification, and enable longitudinal monitoring. The primary objectives of CMR assessment in rTOF patients include to quantify biventricular volumes, function, and mass; characterize regional wall motion abnormalities; assess the RVOT and pulmonary artery; measure pulmonary regurgitant volume and fraction; evaluate tricuspid regurgitation; quantify residual shunts; and assess myocardial viability by detecting focal scarring and/or diffuse fibrosis [60].

Beyond these core objectives, CMR imaging may also contribute to risk prediction. In 2022, Ghonim et al. proposed a risk stratification scheme for adults with rTOF, where late gadolinium enhancement CMR evaluation was added to proven risk factors to identify patients at high risk of death or malignant ventricular arrhythmia [61]. In a recent CMR study by Wald et al., the five-year risk of major adverse cardiovascular events in patients with rTOF was estimated using five variables, including age, diabetes mellitus, RVOT obstruction, atrial and ventricular dimensions [62].

Cardiac computed tomography (CT) represents a valuable imaging modality in cases where patients have indwelling metal, require confirmation of coronary artery anatomy or patency, or are unable to hold their breath during imaging. CT offers superior spatial resolution, making it effective for providing anatomical and functional assessment, identification of coronary artery abnormalities, and pre-procedural modelling [1, 60]. However, its use is limited by radiation exposure and less effective myocardial tissue characterization compared to CMR.

### Telemedicine

Telemedicine constitutes a major advancement in the management of ACHD patients, especially for those experiencing HF. Lifelong specialized follow-up often necessitates travel to distant medical centers, creating significant barriers to care. Telemedicine mitigates geographic disparities by enhancing access to specialized services and reducing travel-related costs. Remote monitoring enables the transmission of clinical data such as weight, blood pressure, and heart rate from patients’ homes to healthcare providers. This capability supports early detection of clinical deterioration, facilitates timely intervention, and decreases hospital admissions, thereby improving patient outcomes [63].

Although the use of telemedicine in the management of ACHD patients is still in the early stages, its ability to offer personalized, specialized care and ensure continuous long-term monitoring makes it a pivotal tool in the ongoing management of these patients. Recent research indicates, indeed, that ACHD patients with chronic HF, including those with TOF, may benefit from a device-based telemedicine program, which reduces hospital admissions and ensures the stabilization of clinical parameters [64].

## Pharmacological strategies

Timely pulmonary valve replacement remains the primary treatment option when indicated [39]. Currently, for patients with TOF and RV dysfunction, there is no widely accepted pharmacological regimen as demonstrated for patients with HF due to LV dysfunction. In symptomatic patients, supportive management with loop diuretics is indicated to alleviate HF symptoms [4, 65].

Several randomized controlled trials have been conducted in patients with TOF, but none have been able to demonstrate a beneficial effect on right ventricular function (Table 1). The APPROPRIATE study, a single-center, double-blind, placebo-controlled study, evaluated the effects of ramipril, an angiotensin-converting enzyme (ACE) inhibitor, in 64 stable patients with rTOF and moderate or severe pulmonary regurgitation. After six months, there was no significant difference in the primary endpoint of right ventricular ejection fraction. Ramipril therapy did not significantly change the severity of pulmonary regurgitation, ventricular volumes, exercise cardiopulmonary performance, or neurohormonal activation. However, a subgroup of patients with restrictive right ventricular physiology experienced a decrease in LV end-systolic volume index and an increase in LV ejection fraction [66].

**Table 1:** Summarize of trials evaluating Pharmacology therapy in patients with repaired tetralogy of Fallot. RV, right ventricle; LV, left ventricle; NS, not significant; NR, not reported; EF, ejection fraction; CMR, cardiac magnetic resonance; NTproBNP, N-terminal pro brain natriuretic peptide; VO2max, maximal oxygen uptake

Study (first author, year, study name)	Study design	Medication	Baseline characteristics	Improve RV function	Improve LV function	Improve pulmonary regurgitation	Improve cardiopulmonary performance
Babu-Narayan 2010 [67] APPROPRIATE (ACE inhibitors for Potential Prevention Of the deleterious effects of Pulmonary Regurgitation in Adults with repaired TEtralogy of Fallot)	Prospective, randomized, single-center, double-blind, placebo-controlled	Ramipril (starting dose 2.5 mg o.d. to target dose 10 mg o.d.)	Adolescent and adult patients with moderate to severe pulmonary regurgitation and without significant pulmonary stenosis	NS	Increase LV EF by CMR in a subgroup of patients with restrictive physiology	NS	NS
Kuprickova 2018 [68]	Post-hoc analysis APPROPRIATE study	Ramipril (starting dose 2.5 mg o.d. to target dose 10 mg o.d.)	Adolescent and adult patients with moderate to severe pulmonary regurgitation and without significant pulmonary stenosis	NR	Yes, increase in diastolic and systolic echocardiographic parameters	NR	NR
Bokma 2017 [69] REDEFINE (Right Ventricular Dysfunction in Tetralogy of Fallot: Inhibition of the Renin-Angiotensin-Aldosterone System)	Prospective, randomized, multicenter, double-blind, placebo-controlled	Losartan (starting dose 50 mg o.d. to target dose 150 mg o.d.)	Adult patients with RV EF < 50% and no severe valvular lesions	NS Improvement RV EF by CMR in a subgroup with nonrestrictive RV and incomplete remodelling	NS	NR	NS
Norozi 2007 [70]	Prospective, randomized, single-center, double-blind, placebo-controlled	Bisoprolol (starting dose 1.25 mg o.d. to target dose 10 mg o.d.)	Adolescent and adult patients with NTproBNP ≥100 pg/ml and VO2max≤25 ml/kg/min	NS	NS	NR	NS

Subsequently, a post-hoc analysis of the APPROPRIATE revealed significantly higher diastolic and systolic echocardiographic parameters of the LV function in patients with TOF after 6 months of ramipril compared to those treated with placebo. Considering these data were partially driven by the progressive decline in measures in the placebo group, these results were interpreted as a stabilizing effect of ramipril therapy [67].

Similarly, the Right Ventricular Dysfunction in Tetralogy of Fallot: Inhibition of the Renin-Angiotensin-Aldosterone System (REDEFINE) study, a multicenter, prospective, randomized, double-blind, placebo-controlled trial, assessed losartan in adults with CHD who had rTOF and right ventricular ejection fraction less than 50% but without severe valvular dysfunction. Losartan therapy did not improve the primary endpoint of right ventricular ejection fraction. Secondary outcomes, including LV ejection fraction, peak aerobic exercise capacity, and N-terminal pro-brain natriuretic peptide levels, also showed no improvement. Notably, losartan was associated with improved ejection fraction in a subgroup of patients with non-restrictive right ventricular physiology and incomplete remodelling [68].

Beta-blocker therapy has also been investigated in this patient population. A prospective, randomized, double-blind, placebo-controlled study evaluated bisoprolol in 33 patients with repaired tetralogy of Fallot who were asymptomatic or mildly symptomatic. The study found no effect on brain natriuretic peptide levels, peak oxygen consumption, or right or LV ejection fraction. NYHA classification also remained unchanged [69].

Emerging therapies, such as angiotensin receptor neprilysin inhibitors (ARNI) and sodium-glucose cotransporter 2 (SGLT2) inhibitors, have demonstrated efficacy in HF management in other populations [70]. To date, no published studies have assessed the efficacy or safety of these agents in patients with rTOF. ARNI inhibits the angiotensin receptor-neprilysin pathway and may offer therapeutic benefit for right ventricular dysfunction. Only a single case report has described its use in TOF [71]. SGLT2 inhibitors are increasingly prescribed for LV failure. Several mechanisms have been proposed for their potential benefit in right ventricular dysfunction, including natriuresis, plasma volume reduction, enhanced tissue oxygen delivery through increased haematocrit, improved endothelium-dependent vasodilation via increased nitric oxide bioavailability, and increased myocardial energy through greater availability of fatty acids, ketone bodies, and branched-chain amino acids. SGLT2 inhibitors may also reduce pulmonary arterial pressure by attenuating pulmonary vascular remodelling [72]. Further research is necessary to determine their therapeutic role in rTOF.

There is a lack of specific data regarding the efficacy of antiarrhythmic medication in TOF. Catheter ablation demonstrated greater effectiveness than antiarrhythmic drugs in managing both atrial and ventricular tachyarrhythmias. Moreover, in patients with moderate and complex CHD, including TOF, initial rhythm control is generally preferred over rate control strategies [73]. Limited research on the effectiveness of various antiarrhythmic agents for ventricular tachyarrhythmias has resulted in inconsistent prescribing practices, leading to a primary management emphasizing arrhythmia suppression through ablation and implantable devices rather than antiarrhythmic pharmacological therapy [74].

## Future directions

Despite significant advances in the understanding and management of rTOF, major challenges remain, particularly the lack of standardized approaches for the timely identification of high-risk patients and the implementation of effective long-term treatments. To address these gaps and improve clinical outcomes, future research should focus on the following key areas:

- *Delineate the natural history of RV dysfunction*: prospective, multicentre studies are needed to clarify the progression of RV dysfunction and identify reliable biomarkers and imaging indicators that predict adverse outcomes. This research should support the development of a dynamic risk stratification models integrating clinical, imaging and genetic factors, enabling pro-active and personalized management.
- *Standardize advanced imaging techniques*: although modern imaging techniques, such as advanced echocardiographic tools and CMR, are essential for risk assessment and stratification, the integration of these tools in clinical practice is hampered by the lack of standardized protocols. New research should focus on the validation of new imaging metrics and creating standardized imaging protocols to ensure data reliability and reproducibility across different clinical scenarios.
- *Evaluate new therapeutic strategies*: given the limited efficacy of current pharmacological therapies, which often fail to improve RV function, it is crucial to rigorously evaluate new therapeutic options. ARNI and SGLT2 inhibitors have demonstrated their benefit for left HF and have promising mechanism of action for RV dysfunction. However, targeted clinical studies to ensure their efficacy and safety in patients with rTOF are lacking. The creation of international registries and targeted clinical trials is essential to fill this gap.
- *Leveraging technological innovation for precision care*: further research is required to evaluate the role of artificial intelligence, computational modelling, digital-twin technology in early identification of ventricular dysfunction and predicting clinical outcome. Similarly, 3D printing and virtual reality deserve further exploration to optimize surgical planning for complex anatomies. The integration of digital technologies and remote monitoring may also enhance long-term surveillance and continuity of care.

## Conclusion

Despite tremendous advancements in surgical repair and clinical management, adult patients with rTOF remain at risk for RV dysfunction due to a complex interplay of anatomical, functional, and electrophysiological complications. The path to optimal management of rTOF require a refined strategy: from a reactive management of complication to a proactive approach based on solid scientific evidence. Implementing the recommendations described above can refine risk stratification, optimize clinical care, and ultimately improve quality of life and long-term outcomes of patients with rTOF.

## Data Availability

No datasets were generated or analysed during the current study.

## References

[1] Moscatelli S, Pergola V, Motta R, Fortuni F, Borrelli N, Sabatino J, Leo I, Avesani M, Montanaro C, Surkova E, Mapelli M, Perrone MA, Working Group on Congenital Heart Disease, Cardiovascular Prevention in Paediatric Age of the Italian Society of Cardiology (SIC) (2023) Multimodality imaging assessment of tetralogy of Fallot: from diagnosis to long-term follow-up. Children 10(11):1747. 10.3390/children10111747 38002838 10.3390/children10111747 PMC10670209

[2] Leonardi B, Perrone M, Calcaterra G, Sabatino J, Leo I, Aversani M, Bassareo PP, Pozza A, Oreto L, Moscatelli S, Borrelli N, Bianco F, Di Salvo G, Working Group on Congenital Heart Disease, Cardiovascular Prevention in Paediatric Age of the Italian Society of Cardiology (SIC) (2024) Repaired tetralogy of Fallot: have we understood the right timing of PVR? J Clin Med 13(9):2682. 10.3390/jcm13092682 38731211 10.3390/jcm13092682 PMC11084704

[3] Verheugt CL, Uiterwaal CS, van der Velde ET, Meijboom FJ, Pieper PG, van Dijk AP, Vliegen HW, Grobbee DE, Mulder BJ (2010) Mortality in adult congenital heart disease. Eur Heart J 31(10):1220–9. 10.1093/eurheartj/ehq032 20207625 10.1093/eurheartj/ehq032

[4] Brida M, Lovrić D, Griselli M, Riesgo Gil F, Gatzoulis MA (2022) Heart failure in adults with congenital heart disease. Int J Cardiol 357:39–45. 10.1016/j.ijcard.2022.03.018 35283250 10.1016/j.ijcard.2022.03.018

[5] den Van Bosch E, Bogers AJJC, Roos-Hesselink JW, van Dijk APJ, van Wijngaarden MHEJ, Boersma E, Nijveld A, Luijten LWG, Tanke R, Koopman LP, Helbing WA (2020) Long-term follow-up after transatrial-transpulmonary repair of tetralogy of Fallot: influence of timing on outcome. Eur J Cardiothorac Surg 57(4):635–643. 10.1093/ejcts/ezz331 31872208 10.1093/ejcts/ezz331 PMC7078865

[6] Avesani M, Jalal Z, Friedberg MK, Villemain O, Venet M, Di Salvo G, Thambo JB, Iriart X (2024) Adverse remodelling in tetralogy of Fallot: from risk factors to imaging analysis and future perspectives. Hellenic J Cardiol 75:48–59. 10.1016/j.hjc.2023.07.008 37495104 10.1016/j.hjc.2023.07.008

[7] Frigiola A, Redington AN, Cullen S, Vogel M (2004) Pulmonary regurgitation is an important determinant of right ventricular contractile dysfunction in patients with surgically repaired tetralogy of Fallot. Circulation 110(11 Suppl 1):II153-7. 10.1161/01.CIR.0000138397.60956.c2 15364855 10.1161/01.CIR.0000138397.60956.c2

[8] Geva T (2011) Repaired tetralogy of Fallot: the roles of cardiovascular magnetic resonance in evaluating pathophysiology and for pulmonary valve replacement decision support. J Cardiovasc Magn Reson 13(1):9. 10.1186/1532-429X-13-9 21251297 10.1186/1532-429X-13-9 PMC3036629

[9] Alipour Symakani RS, van Genuchten WJ, Zandbergen LM, Henry S, Taverne YJHJ, Merkus D, Helbing WA, Bartelds B (2023) The right ventricle in tetralogy of Fallot: adaptation to sequential loading. Front Pediatr 11:1098248. 10.3389/fped.2023.1098248. (**PMID: 37009270; PMCID: PMC10061113**) 37009270 10.3389/fped.2023.1098248 PMC10061113

[10] Atallah-Yunes NH, Kavey RE, Bove EL, Smith FC, Kveselis DA, Byrum CJ, Gaum WE (1996) Postoperative assessment of a modified surgical approach to repair of tetralogy of Fallot. Long-term follow-up. Circulation. ;94(9 Suppl):II22-6. PMID: 8901714 8901714

[11] Latus H, Hachmann P, Gummel K, Khalil M, Yerebakan C, Bauer J, Schranz D, Apitz C (2015) Impact of residual right ventricular outflow tract obstruction on biventricular strain and synchrony in patients after repair of tetralogy of Fallot: a cardiac magnetic resonance feature tracking study. Eur J Cardiothorac Surg 48(1):83–90. 10.1093/ejcts/ezu396 25378364 10.1093/ejcts/ezu396

[12] Latus H, Gummel K, Rupp S, Valeske K, Akintuerk H, Jux C, Bauer J, Schranz D, Apitz C (2013) Beneficial effects of residual right ventricular outflow tract obstruction on right ventricular volume and function in patients after repair of tetralogy of Fallot. Pediatr Cardiol 34(2):424–430. 10.1007/s00246-012-0476-4. (**Epub 2012 Aug 23. PMID: 22915139**) 22915139 10.1007/s00246-012-0476-4

[13] Latus H, Stammermann J, Voges I, Waschulzik B, Gutberlet M, Diller GP, Schranz D, Ewert P, Beerbaum P, Kühne T, Sarikouch S (2022) Impact of right ventricular pressure load after repair of tetralogy of Fallot. J Am Heart Assoc 11(7):e022694. 10.1161/JAHA.121.022694 35301850 10.1161/JAHA.121.022694 PMC9075442

[14] Valente AM, Gauvreau K, Assenza GE, Babu-Narayan SV, Schreier J, Gatzoulis MA, Groenink M, Inuzuka R, Kilner PJ, Koyak Z, Landzberg MJ, Mulder B, Powell AJ, Wald R, Geva T (2014) Contemporary predictors of death and sustained ventricular tachycardia in patients with repaired tetralogy of Fallot enrolled in the INDICATOR cohort. Heart 100(3):247–253. 10.1136/heartjnl-2013-304958. (**Epub 2013 Oct 31. PMID: 24179163; PMCID: PMC3913216**) 24179163 10.1136/heartjnl-2013-304958 PMC3913216

[15] DiLorenzo M, Hwang WT, Goldmuntz E, Ky B, Mercer-Rosa L (2018) Diastolic dysfunction in tetralogy of Fallot: comparison of echocardiography with catheterization. Echocardiography 35(10):1641–1648. 10.1111/echo.14113 30105757 10.1111/echo.14113 PMC6205242

[16] Egbe AC, Pellikka PA, Miranda WR, Bonnichsen C, Reddy YNV, Borlaug BA, Connolly HM (2020) Echocardiographic predictors of severe right ventricular diastolic dysfunction in tetralogy of Fallot: relations to patient outcomes. Int J Cardiol 306:49–55 32145939 10.1016/j.ijcard.2020.02.067 PMC7297267

[17] Gatzoulis MA, Clark AL, Cullen S, Newman CGH, Redington AN (1995) Right ventricular diastolic function 15 to 35 years after repair of tetralogy of Fallot: restrictive physiology predicts superior exercise performance. Circulation 91:1775–1781 7882487 10.1161/01.cir.91.6.1775

[18] Kutty S, Valente AM, White MT et al (2018) Usefulness of pulmonary arterial end-diastolic forward flow late after tetralogy of Fallot repair to predict a “restrictive” right ventricle. Am J Cardiol 121:1380–1386 29678339 10.1016/j.amjcard.2018.02.025

[19] den Van Eynde J, Derdeyn E, Schuermans A et al (2022) End-diastolic forward flow and restrictive physiology in repaired tetralogy of Fallot: a systematic review and meta-analysis. J Am Heart Assoc 11(7):e024036 35301867 10.1161/JAHA.121.024036 PMC9075485

[20] Ait Ali L, Celi S, Festa P, Parameters? (2024) JACC Asia 4(12):925–927. 10.1016/j.jacasi.2024.10.018PMID: 39803002; PMCID: PMC11712012 39803002 10.1016/j.jacasi.2024.10.018 PMC11712012

[21] Cheung YF, Yu CKM, So EKF, Li VWY, Wong WHS (2019) Atrial strain imaging after repair of tetralogy of Fallot: a systematic review. Ultrasound Med Biol 45(8):1896–1908. 10.1016/j.ultrasmedbio.2019.04.032 31153717 10.1016/j.ultrasmedbio.2019.04.032

[22] Avesani M, Borrelli N, Krupickova S, Sabatino J, Donne GD, Ibrahim A, Piccinelli E, Josen M, Michielon G, Fraisse A, Iliceto S, Di Salvo G (2020) Echocardiography and cardiac magnetic resonance in children with repaired tetralogy of Fallot: new insights in cardiac mechanics and exercise capacity. Int J Cardiol 321:144–149. 10.1016/j.ijcard.2020.07.026 32702408 10.1016/j.ijcard.2020.07.026

[23] Luijnenburg SE, Peters RE, van der Geest RJ, Moelker A, Roos-Hesselink JW, de Rijke YB, Mulder BJ, Vliegen HW, Helbing WA (2013) Abnormal right atrial and right ventricular diastolic function relate to impaired clinical condition in patients operated for tetralogy of Fallot. Int J Cardiol 167(3):833–9. 10.1016/j.ijcard.2012.02.011 22390967 10.1016/j.ijcard.2012.02.011

[24] Ait-ALi L, Marrone C, Salvadori S, Federici D, Pak V, Arcieri L, Passino C, Santoro G, Festa P (2020) Impact of right atrium dimension on adverse outcome after pulmonary valve replacement in repaired tetralogy of Fallot patients. Int J Cardiovasc Imaging 36(10):1973–1982. 10.1007/s10554-020-01891-9. (**Epub 2020 May 27. PMID: 32462447**) 32462447 10.1007/s10554-020-01891-9

[25] Rebergen SA, Chin JG, Ottenkamp J, van der Wall EE, de Roos A (1993) Pulmonary regurgitation in the late postoperative follow-up of tetralogy of Fallot. Volumetric quantitation by nuclear magnetic resonance velocity mapping. Circulation 88(5 Pt 1):2257–66. 10.1161/01.cir.88.5.2257 8222120 10.1161/01.cir.88.5.2257

[26] Xie M, Li Y, Cheng TO, Wang X, Dong N, Nie X et al (2015) The effect of right ventricular myocardial remodeling on ventricular function as assessed by two-dimensional speckle tracking echocardiography in patients with tetralogy of fallot: a single center experience from China. Int J Cardiol 178:300–307. 10.1016/j.ijcard.2014.10.02725453412 25453412 10.1016/j.ijcard.2014.10.027

[27] Sanchez-Quintana D, Anderson RH, Ho SY (1996) Ventricular myoarchitecture in tetralogy of Fallot. Heart 76(3):280–286. 10.1136/hrt.76.3.280. (**PMID: 8868990; PMCID: PMC484521**) 8868990 10.1136/hrt.76.3.280 PMC484521

[28] Babu-Narayan SV, Kilner PJ, Li W, Moon JC, Goktekin O, Davlouros PA, Khan M, Ho SY, Pennell DJ, Gatzoulis MA (2006) Ventricular fibrosis suggested by cardiovascular magnetic resonance in adults with repaired tetralogy of Fallot and its relationship to adverse markers of clinical outcome. Circulation 113(3):405–13. 10.1161/CIRCULATIONAHA.105.548727 16432072 10.1161/CIRCULATIONAHA.105.548727

[29] Wald RM, Haber I, Wald R, Valente AM, Powell AJ, Geva T (2009) Effects of regional dysfunction and late gadolinium enhancement on global right ventricular function and exercise capacity in patients with repaired tetralogy of Fallot. Circulation 119(10):1370–1377. 10.1161/CIRCULATIONAHA.108.816546. (**Epub 2009 Mar 2. PMID: 19255342; PMCID: PMC2764308**) 19255342 10.1161/CIRCULATIONAHA.108.816546 PMC2764308

[30] Ghonim S, Ernst S, Keegan J, Giannakidis A, Spadotto V, Voges I, Smith GC, Boutsikou M, Montanaro C, Wong T, Ho SY, McCarthy KP, Shore DF, Dimopoulos K, Uebing A, Swan L, Li W, Pennell DJ, Gatzoulis MA, Babu-Narayan SV (2020) Three-dimensional late gadolinium enhancement cardiovascular magnetic resonance predicts inducibility of ventricular tachycardia in adults with repaired tetralogy of Fallot. Circ Arrhythm Electrophysiol 13(11):e008321. 10.1161/CIRCEP.119.008321 33022183 10.1161/CIRCEP.119.008321

[31] Chen CA, Dusenbery SM, Valente AM, Powell AJ, Geva T (2016) Myocardial ECV fraction assessed by CMR is associated with type of hemodynamic load and arrhythmia in repaired tetralogy of Fallot. JACC Cardiovasc Imaging 9(1):1–10. 10.1016/j.jcmg.2015.09.011 26684969 10.1016/j.jcmg.2015.09.011

[32] Shiina Y, Taniguchi K, Nagao M, Takahashi T, Niwa K, Kawakubo M, Inai K (2020) The relationship between extracellular volume fraction in symptomatic adults with tetralogy of Fallot and adverse cardiac events. J Cardiol 75(4):424–431. 10.1016/j.jjcc.2019.09.009 31615743 10.1016/j.jjcc.2019.09.009

[33] Cochet H, Iriart X, Allain-Nicolaï A, Camaioni C, Sridi S, Nivet H, Fournier E, Dinet ML, Jalal Z, Laurent F, Montaudon M, Thambo JB (2019) Focal scar and diffuse myocardial fibrosis are independent imaging markers in repaired tetralogy of Fallot. Eur Heart J Cardiovasc Imaging 20(9):990–1003. 10.1093/ehjci/jez068 30993335 10.1093/ehjci/jez068 PMC6704392

[34] Cools B, Nagaraju CK, Vandendriessche K, van Puyvelde J, Youness M, Roderick HL, Gewillig M, Sipido K, Claus P, Rega F (2022) Reversal of right ventricular remodeling after correction of pulmonary regurgitation in tetralogy of Fallot. JACC Basic Transl Sci 8(3):301–315. 10.1016/j.jacbts.2022.09.008 37034286 10.1016/j.jacbts.2022.09.008 PMC10077151

[35] Krieger EV, Zeppenfeld K, DeWitt ES, Duarte VE, Egbe AC, Haeffele C, Lin KY, Robinson MR, Sillman C, Upadhyay S, American Heart Association Adults With Congenital Heart Disease Committee of the Council on Lifelong Congenital Heart Disease and Heart Health in the Young and Council on Clinical Cardiology (2022) Arrhythmias in repaired tetralogy of Fallot: a scientific statement from the American Heart Association. Circ Arrhythm Electrophysiol 15(11):e000084. 10.1161/HAE.0000000000000084 36263773 10.1161/HAE.0000000000000084

[36] Khairy P, Aboulhosn J, Gurvitz MZ, Opotowsky AR, Mongeon FP, Kay J, Valente AM, Earing MG, Lui G, Gersony DR, Cook S, Ting JG, Nickolaus MJ, Webb G, Landzberg MJ, Broberg CS, Alliance for Adult Research in Congenital Cardiology (AARCC) (2010) Arrhythmia burden in adults with surgically repaired tetralogy of Fallot: a multi-institutional study. Circulation 122(9):868–75. 10.1161/CIRCULATIONAHA.109.928481 20713900 10.1161/CIRCULATIONAHA.109.928481

[37] Khairy P (2007) Programmed ventricular stimulation for risk stratification in patients with tetralogy of Fallot: a Bayesian perspective. Nat Clin Pract Cardiovasc Med 4(6):292–3. 10.1038/ncpcardio0882 17522719 10.1038/ncpcardio0882

[38] Vehmeijer JT, Koyak Z, Leerink JM, Zwinderman AH, Harris L, Peinado R, Oechslin EN, Robbers-Visser D, Groenink M, Boekholdt SM, de Winter RJ, Oliver JM, Bouma BJ, Budts W, Van Gelder IC, Mulder BJM, de Groot JR (2021) Identification of patients at risk of sudden cardiac death in congenital heart disease: the prospective study on implantable cardioverter defibrillator therapy and sudden cardiac death in adults with congenital heart disease (PREVENTION-ACHD). Heart Rhythm 18(5):785–792. 10.1016/j.hrthm.2021.01.009Epub 2021 Jan 16. PMID: 33465514 33465514 10.1016/j.hrthm.2021.01.009

[39] Baumgartner H, De Backer J, Babu-Narayan SV, Budts W, Chessa M, Diller GP, Lung B, Kluin J, Lang IM, Meijboom F, Moons P, Mulder BJM, Oechslin E, Roos-Hesselink JW, Schwerzmann M, Sondergaard L, Zeppenfeld K (2021) 2020 ESC guidelines for the management of adult congenital heart disease. Eur Heart J 42(6):563–645. 10.1093/eurheartj/ehaa554 32860028 10.1093/eurheartj/ehaa554

[40] Alborikan S, Pandya B, Von Klemperer K, Walker F, Cullen S, Badiani S, Bhattacharyya S, Lloyd G (2020) Cardiopulmonary exercise test (CPET) in patients with repaired Tetralogy of Fallot (rTOF): a systematic review. Int J Cardiol Congenit Heart Dis 1:100050. 10.1016/j.ijcchd.2020.100050 10.1016/j.ijcchd.2023.100483 PMC11657782 39712984

[41] Babu-Narayan SV, Diller GP, Gheta RR, Bastin AJ, Karonis T, Li W, Pennell DJ, Uemura H, Sethia B, Gatzoulis MA, Shore DF (2014) Clinical outcomes of surgical pulmonary valve replacement after repair of tetralogy of Fallot and potential prognostic value of preoperative cardiopulmonary exercise testing. Circulation 129(1):18–27 (**Epub 2013 Oct 21. PMID: 24146254**) 24146254 10.1161/CIRCULATIONAHA.113.001485

[42] Leonardi B, Cifra B (2023) The role of cardiopulmonary testing to risk stratify tetralogy of Fallot patients. CJC Pediatric and Congenital Heart Disease 2(6Part A):314–321. 10.1016/j.cjcpc.2023.10.007 38161674 10.1016/j.cjcpc.2023.10.007 PMC10755826

[43] Shafer KM, Opotowsky AR, Rhodes J (2018) Exercise testing and spirometry as predictors of mortality in congenital heart disease: contrasting Fontan physiology with repaired tetralogy of Fallot: XXXX. Congenit Heart Dis 13(6):903–910. 10.1111/chd.12661 30216689 10.1111/chd.12661

[44] Diller GP, Dimopoulos K, Okonko D, Uebing A, Broberg CS, Babu-Narayan S, Bayne S, Poole-Wilson PA, Sutton R, Francis DP, Gatzoulis MA (2006) Heart rate response during exercise predicts survival in adults with congenital heart disease. J Am Coll Cardiol 48(6):1250–6. 10.1016/j.jacc.2006.05.051 16979014 10.1016/j.jacc.2006.05.051

[45] Diller GP, Dimopoulos K, Okonko D, Li W, Babu-Narayan SV, Broberg CS, Johansson B, Bouzas B, Mullen MJ, Poole-Wilson PA, Francis DP, Gatzoulis MA (2005) Exercise intolerance in adult congenital heart disease: comparative severity, correlates, and prognostic implication. Circulation 112(6):828–835 Epub 2005 Aug 1. PMID: 16061735 16061735 10.1161/CIRCULATIONAHA.104.529800

[46] Müller J, Hager A, Diller GP, Derrick G, Buys R, Dubowy KO, Takken T, Orwat S, Inuzuka R, Vanhees L, Gatzoulis M, Giardini A (2015) Peak oxygen uptake, ventilatory efficiency and QRS-duration predict event free survival in patients late after surgical repair of tetralogy of Fallot. Int J Cardiol 196:158–64. 10.1016/j.ijcard.2015.05.174 26114442 10.1016/j.ijcard.2015.05.174

[47] Borrelli N, Di Salvo G, Sabatino J, Ibrahim A, Avesani M, Sirico D, Josen M, Penco M, Fraisse A, Michielon G (2020) Serial changes in longitudinal strain are associated with outcome in children with hypoplastic left heart syndrome. Int J Cardiol 317:56–62. 10.1016/j.ijcard.2020.03.085 32505372 10.1016/j.ijcard.2020.03.085

[48] Iriart X, Montaudon M, Lafitte S, Chabaneix J, Réant P, Balbach T, Houle H, Laurent F, Thambo JB (2009) Right ventricle three-dimensional echography in corrected tetralogy of fallot: accuracy and variability. Eur J Echocardiogr 10(6):784–92. 10.1093/ejechocard/jep071 19502620 10.1093/ejechocard/jep071

[49] Bidviene J, Muraru D, Maffessanti F, Ereminiene E, Kovács A, Lakatos B, Vaskelyte JJ, Zaliunas R, Surkova E, Parati G, Badano LP (2021) Regional shape, global function and mechanics in right ventricular volume and pressure overload conditions: a three-dimensional echocardiography study. Int J Cardiovasc Imaging 37(4):1289–1299. 10.1007/s10554-020-02117-8Epub 2021 Jan 3. PMID: 33389362; PMCID: PMC8026459 33389362 10.1007/s10554-020-02117-8 PMC8026459

[50] Scherptong RW, Mollema SA, Blom NA, Kroft LJ, de Roos A, Vliegen HW, van der Wall EE, Bax JJ, Holman ER (2009) Right ventricular peak systolic longitudinal strain is a sensitive marker for right ventricular deterioration in adult patients with tetralogy of Fallot. Int J Cardiovasc Imaging 25(7):669–76. 10.1007/s10554-009-9477-7 19642012 10.1007/s10554-009-9477-7 PMC2729418

[51] Arroyo-Rodríguez C, Fritche-Salazar JF, Posada-Martínez EL, Arías-Godínez JA, Ortiz-León XA, Calvillo-Arguelles O, Ruiz-Esparza ME, Sandoval JP, Sierra-Lara D, Araiza-Garaygordobil D, Picano E, Rodríguez-Zanella H (2020) Right ventricular free wall strain predicts functional capacity in patients with repaired Tetralogy of Fallot. Int J Cardiovasc Imaging 36(4):595–604. 10.1007/s10554-019-01753-z 31894525 10.1007/s10554-019-01753-z

[52] van Grootel RWJ, van den Bosch AE, Baggen VJM, Menting ME, Baart SJ, Cuypers JAAE, Witsenburg M, Roos-Hesselink JW (2019) The prognostic value of myocardial deformation in adult patients with corrected Tetralogy of Fallot. J Am Soc Echocardiogr 32(7):866-875.e2. 10.1016/j.echo.2019.03.014 31064677 10.1016/j.echo.2019.03.014

[53] Sabatino J, Leo I, Strangio A, Bella S, Borrelli N, Avesani M, Josen M, Paredes J, Piccinelli E, Sirico D, Pergola V, Fraisse A, De Rosa S, Indolfi C, Di Salvo G (2022) Echocardiographic normal reference ranges for non-invasive myocardial work parameters in pediatric age: results from an international multi-center study. Front Cardiovasc Med 9:792622. 10.3389/fcvm.2022.792622 35548421 10.3389/fcvm.2022.792622 PMC9081714

[54] Butcher SC, Feloukidis C, Kamperidis V, Yedidya I, Stassen J, Fortuni F, Vrana E, Mouratoglou SA, Boutou A, Giannakoulas G, Playford D, Ajmone Marsan N, Bax JJ, Delgado V (2022) Right ventricular myocardial work characterization in patients with pulmonary hypertension and relation to invasive hemodynamic parameters and outcomes. Am J Cardiol 177:151–161. 10.1016/j.amjcard.2022.04.058 35691706 10.1016/j.amjcard.2022.04.058

[55] Dragulescu A, Friedberg MK, Grosse-Wortmann L, Redington A, Mertens L (2014) Effect of chronic right ventricular volume overload on ventricular interaction in patients after tetralogy of Fallot repair. J Am Soc Echocardiogr 27(8):896–902. 10.1016/j.echo.2014.04.012 24846007 10.1016/j.echo.2014.04.012

[56] Li SN, Yu W, Lai CT, Wong SJ, Cheung YF (2013) Left ventricular mechanics in repaired tetralogy of Fallot with and without pulmonary valve replacement: analysis by three-dimensional speckle tracking echocardiography. PLoS One 8(11):e78826. 10.1371/journal.pone.0078826. (**PMID: 24223166; PMCID: PMC3819374**) 24223166 10.1371/journal.pone.0078826 PMC3819374

[57] Yamada M, Takahashi K, Kobayashi M, Yazaki K, Takayasu H, Akimoto K, Kishiro M, Inage A, Yoshikawa T, Park IS, Nakanishi K, Kawasaki S, Shimizu T (2017) Mechanisms of left ventricular dysfunction assessed by layer-specific strain analysis in patients with repaired Tetralogy of Fallot. Circ J 81(6):846–854. 10.1253/circj.CJ-16-1162 28260735 10.1253/circj.CJ-16-1162

[58] Vautier M, Mulet B, Macquaire C, Cousergue C, André CO, Maragnes P, Ollitrault P, Labombarda F (2023) Abnormal left atrial compliance is associated with a history of life-threatening arrhythmia in corrected tetralogy of Fallot. Front Cardiovasc Med 10:1161017. 10.3389/fcvm.2023.1161017. (**PMID: 37180807; PMCID: PMC10169587**) 37180807 10.3389/fcvm.2023.1161017 PMC10169587

[59] Egbe AC, Miranda WR, Madhavan M, Abozied O, Younis A, Ahmed MH, Connolly HM, Deshmukh AJ (2024) Corrigendum to “Right atrial dysfunction is associated with atrial arrhythmias in adults with repaired tetralogy of Fallot” [American Heart Journal Volume 263 (2023)141–150]. Am Heart J 275:191. 10.1016/j.ahj.2024.07.010 37271358 10.1016/j.ahj.2023.05.018

[60] Geva T, Wald RM, Bucholz E, Cnota JF, McElhinney DB, Mercer-Rosa LM, Mery CM, Miles AL, Moore J, American Heart Association Council on Lifelong Congenital Heart Disease and Heart Health in the Young; Council on Cardiovascular Surgery and Anesthesia; Council on Clinical Cardiology; and Council on Cardiovascular and Stroke Nursing (2024) Long-term management of right ventricular outflow tract dysfunction in repaired tetralogy of Fallot: a scientific statement from the American Heart Association. Circulation 150(25):e689–e707. 10.1161/CIR.0000000000001291. (**Epub 2024 Nov 21. PMID: 39569497**) 39569497 10.1161/CIR.0000000000001291

[61] Ghonim S, Gatzoulis MA, Ernst S, Li W, Moon JC, Smith GC, Heng EL, Keegan J, Ho SY, McCarthy KP, Shore DF, Uebing A, Kempny A, Alpendurada F, Diller GP, Dimopoulos K, Pennell DJ, Babu-Narayan SV (2022) Predicting survival in repaired tetralogy of Fallot: a lesion-specific and personalized approach. JACC Cardiovasc Imaging 15(2):257–268. 10.1016/j.jcmg.2021.07.026 34656466 10.1016/j.jcmg.2021.07.026 PMC8821017

[62] Wald RM, Tomlinson G, Caldarone CA, Dahdah N, Dallaire F, Drolet C, Farkouh ME, Grewal J, Hancock-Friesen C, Hickey EJ, Karur GR, Keir M, Kovacs AH, Leonardi B, McCrindle BW, Nadeem SN, Ng MY, Samuel M, Shah A, Tham EB, Therrien J, Van De Bruaene A, Vonder Muhll IF, Warren AE, Yamamura K, Khairy P (2025) Outcome prediction after tetralogy of Fallot repair: a prospective clinical and cardiovascular magnetic resonance study. J Am Heart Assoc 14(8):e039006. 10.1161/JAHA.124.039006 40207483 10.1161/JAHA.124.039006 PMC12132828

[63] Borrelli N, Grimaldi N, Papaccioli G, Fusco F, Palma M, Sarubbi B (2023) Telemedicine in adult congenital heart disease: usefulness of digital health technology in the assistance of critical patients. Int J Environ Res Public Health 20(10):5775. 10.3390/ijerph20105775 37239504 10.3390/ijerph20105775 PMC10218523

[64] Borrelli N, Grimaldi N, Fusco F, Orlando A, Palma M, Boccia MC, Bassolino S, Iervolino A, Sarubbi B (2025) Feasibility and effectiveness of telemedicine for adult patients with congenital heart disease: a one-year single-center experience-based study. International Journal of Cardiology Congenital Heart Disease 20:100582. 10.1016/j.ijcchd.2025.100582 40475705 10.1016/j.ijcchd.2025.100582 PMC12140027

[65] Carazo M (2024) Medical therapy for heart failure in adult congenital heart disease patients. Struct Heart 8(4):100297. 10.1016/j.shj.2024.100297 39100588 10.1016/j.shj.2024.100297 PMC11294834

[66] Babu-Narayan SV, Uebing A, Davlouros PA, Kemp M, Davidson S, Dimopoulos K, Bayne S, Pennell DJ, Gibson DG, Flather M, Kilner PJ, Li W, Gatzoulis MA (2012) Randomised trial of ramipril in repaired tetralogy of Fallot and pulmonary regurgitation: the APPROPRIATE study (Ace inhibitors for Potential PRevention Of the deleterious effects of Pulmonary Regurgitation In Adults with repaired TEtralogy of Fallot). Int J Cardiol 154(3):299–305. 10.1016/j.ijcard.2010.09.057 20970202 10.1016/j.ijcard.2010.09.057

[67] Krupickova S, Li W, Cheang MH, Rigby ML, Uebing A, Davlouros P, Dimopoulos K, Di Salvo G, Fraisse A, Swan L, Alonso-Gonzalez R, Kempny A, Pennell DJ, Senior R, Gatzoulis MA, Babu-Narayan SV (2018) Ramipril and left ventricular diastolic function in stable patients with pulmonary regurgitation after repair of tetralogy of Fallot. Int J Cardiol 272:64–69. 10.1016/j.ijcard.2018.07.132 30153993 10.1016/j.ijcard.2018.07.132

[68] Bokma JP, Winter MM, van Dijk AP, Vliegen HW, van Melle JP, Meijboom FJ, Post MC, Berbee JK, Boekholdt SM, Groenink M, Zwinderman AH, Mulder BJM, Bouma BJ (2018) Effect of losartan on right ventricular dysfunction: results from the double-blind, randomized REDEFINE trial (Right ventricular dysfunction in tetralogy of fallot: Inhibition of the Renin-Angiotensin-Aldosterone System) in adults with repaired tetralogy of fallot. Circulation 137(14):1463–1471. 10.1161/CIRCULATIONAHA.117.031438. (**Epub 2017 Dec 8. PMID: 29222139**) 29222139 10.1161/CIRCULATIONAHA.117.031438

[69] Norozi K, Bahlmann J, Raab B, Alpers V, Arnhold JO, Kuehne T, Klimes K, Zoege M, Geyer S, Wessel A, Buchhorn R (2007) A prospective, randomized, double-blind, placebo controlled trial of beta-blockade in patients who have undergone surgical correction of tetralogy of Fallot. Cardiol Young 17(4):372–9. 10.1017/S1047951107000844 17572925 10.1017/S1047951107000844

[70] McDonagh TA, Metra M, Adamo M, Gardner RS, Baumbach A, Böhm M, Burri H, Butler J, Čelutkienė J, Chioncel O, Cleland JGF, Crespo-Leiro MG, Farmakis D, Gilard M, Heymans S, Hoes AW, Jaarsma T, Jankowska EA, Lainscak M, Lam CSP, Lyon AR, McMurray JJV, Mebazaa A, Mindham R, Muneretto C, Francesco Piepoli M, Price S, Rosano GMC, Ruschitzka F, Skibelund AK (2023) 2023 focused update of the 2021 ESC guidelines for the diagnosis and treatment of acute and chronic heart failure. Eur Heart J 44(37):3627–3639. 10.1093/eurheartj/ehad195 37622666 10.1093/eurheartj/ehad195

[71] Appadurai V, Thoreau J, Malpas T, Nicolae M (2020) Sacubitril/valsartan in adult congenital heart disease patients with chronic heart failure - a single centre case series and call for an international registry. Heart Lung Circ 29(1):137–141. 10.1016/j.hlc.2018.12.003 30686641 10.1016/j.hlc.2018.12.003

[72] Cinar T, Saylik F, Cicek V, Pay L, Khachatryan A, Alejandro J, Erdem A, Hayiroglu MI (2024) Effects of SGLT2 inhibitors on right ventricular function in heart failure patients: updated meta-analysis of the current literature. Kardiol Pol 82(4):416–422. 10.33963/v.phj.100199 38638090 10.33963/v.phj.100199

[73] Egbe AC, Miranda WR, Ammash NM, Ananthaneni S, Sandhyavenu H, Farouk Abdelsamid M, Yogeswaran V, Kapa S, Fatola A, Kothapalli S, Connolly HM (2019) Atrial fibrillation therapy and heart failure hospitalization in adults with tetralogy of Fallot. JACC: Clinical Electrophysiology 5(5):618–625. 10.1016/j.jacep.2019.01.005 31122385 10.1016/j.jacep.2019.01.005

[74] Zoni Berisso M, Drago F, Battaglia A, Mariucci E, Mirizzi G, Vignati G, Sarubbi B (2023) Gestione delle aritmie cardiache dopo correzione chirurgica della tetralogia di Fallot: revisione della letteratura [Management of postoperative arrhythmias in the tetralogy of Fallot: a literature review]. G Ital Cardiol (Rome) 24(11):893–910. 10.1714/4129.41232 37901980 10.1714/4129.41232

